# Clinician awareness, testing, and treatment for lipoprotein(a): Results from a large US national survey

**DOI:** 10.1016/j.ajpc.2025.101388

**Published:** 2025-12-20

**Authors:** Nathan D. Wong, Yihang Fan, Wenjun Fan, Jonathan H Ward, Belinda Schludi, Xingdi Hu

**Affiliations:** a Mary and Steve Wen Cardiovascular Division and Center for Global Cardiometabolic Health and Nutrition, University of California, Irvine; b Novartis Pharmaceuticals Corporation

**Keywords:** Dyslipidemia, Lipoprotein(a), Prevention, Cardiovascular disease

## Abstract

**Background:**

Data are limited regarding national clinician awareness, testing, and treatment of lipoprotein(a) [Lp(a)]. We conducted a national survey of US clinicians to investigate these issues.

**Methods:**

An internet-based survey of awareness, testing and treatment of Lp(a) was administered by a medical survey company to clinicians who have been in practice ≥5 years in the US or its territories.

**Results:**

2002 clinicians completed the survey: 47 % were primary care, 35 % cardiology, 9 % endocrinology, and 9 % neurology. 28 % were female, 24 % Asian, 4 % Hispanic, and 3 % Black. *Awareness:* 81 % of respondents agreed Lp(a) is a significant risk driver for cardiovascular disease (CVD). 77 % and 75 % agreed knowing Lp(a) would help in risk stratification and increase patient engagement, respectively. *Testing:* 41 % of respondents agreed with universal testing of Lp(a). Most agreed Lp(a) should be measured in those with premature (73 %), family history of premature (71 %), or recurrent CVD events (68 %). *Treatment:* 77 % reported having CVD outcome data were felt to be very important for a new therapy, followed by long-term efficacy/safety data (69 %), real-world data (53 %), magnitude of Lp(a) reduction (21 %), dosing frequency (17 %), and mechanism of action (12 %). Clinicians reported being most likely to consider prescribing Lp(a)-targeted therapy with proven CVD benefit among patients with premature (47 %) or recurrent (51 %) CVD events.

**Conclusion:**

Most clinicians agree knowing the Lp(a) level can improve risk assessment and patient engagement. Patients with premature or recurrent CVD events are most likely to be targeted for Lp(a) testing and for prescribing possible future Lp(a)-targeted therapies.

## Graphical abstract

**Figure:**
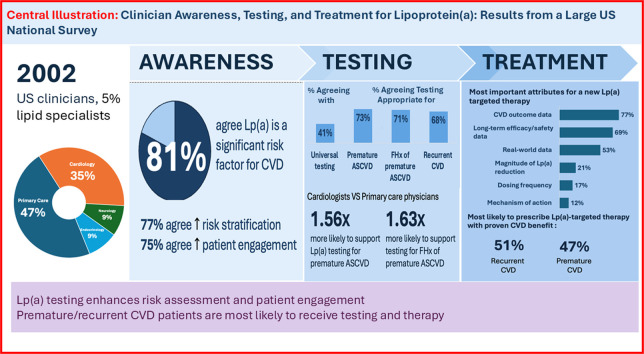

A wealth of data from epidemiologic, genome-wide association studies, and Mendelian randomization studies document lipoprotein(a) [Lp(a)] to be a causal risk factor for atherosclerotic cardiovascular disease (ASCVD) [1,2]. Lp(a) levels have been recently documented to have a linear relationship with cardiovascular event risk in a large meta-analysis with a mean follow-up of 3.0 years [3] for both baseline as well as on-statin Lp(a) levels. We have also previously demonstrated Lp(a) to strongly predict future ASCVD events in statin-treated individuals with ASCVD [4] and to be the strongest factor predicting future ASCVD events in persons with known ASCVD [5]. Moreover, our recent pooling of five major US cohort studies comprising over 27,000 adults demonstrates consistently strong associations of higher levels of Lp(a) in predicting ASCVD events, especially for the prediction of myocardial infarction and revascularization and with stronger associations in persons with vs. without diabetes [6].

Recommendations and guidelines from the American Heart Association and other societies consider Lp(a) could be measured in persons with a personal or family history of ASCVD [7,8], in women when hypercholesterolemia is present [8], with more recent recommendations from the US National Lipid Association recommending testing at least once in all adults [9]. Moreover, most societies are aligned on recognizing an Lp(a) of ≥50 mg/dL or >125 nmol/L as elevated warranting initiation or intensification of risk reducing therapy such as statins. It has also been noted that more widespread population screening be considered once measurement issues are further addressed [7].

Recently published data document fewer than 1 % of the US population have Lp(a) tested. Hu et al. [10] documented from the Optum Clinformatics Data Mart database that among patients with prior cholesterol-lowering medication and laboratory data only 0.7 % of secondary prevention and 0.6 % of primary prevention patients had an Lp(a) test done. And in a study of the University of California Health data, Bhatia et al. [11] showed testing rates of only 0.3 % overall and ≤4 % in those with a personal or family history of ASCVD. In another recently published study utilizing data from 11 US healthcare systems participating in the National Patient-centered Clinical Research Network (PCORnet), patients tested for Lp(a) (compared to those with LDL-C tests without Lp(a) measures) more frequently had prevalent ASCVD and multiple prior CVD events. Moreover, elevated Lp(a) was associated with a greater likelihood of subsequent lipid lowering therapy initiation as well as cardiovascular hospitalizations [12]. The use of pre-procedure order sets and electronic health record messaging has been shown to significantly increase Lp(a) testing overall and especially in those with aortic stenosis or a TAVR procedure [13]. Finally, in a study where providers were randomized to receiving pre-appointment reminders for Lp(a) testing recommended to appropriate patients, those getting such pre-appointment messages vs. not getting such messages were more likely to result in Lp(a) orders and with more tests performed [14].

Lack of knowledge of Lp(a) as a significant ASCVD risk driver, how to manage patients with elevated Lp(a), or presence of reasons/barriers may contribute to the lack of Lp(a) testing. We conducted a nationwide survey of US clinicians to better understand the contemporary perception of Lp(a) as a risk driver for ASCVD, Lp(a) testing practice, barriers to testing, management of patients with elevated Lp(a), and expectations of future treatments for Lp(a) lowering.

## 1 Methods

### 1.1 Sample Selection

We conducted a non-interventional, cross-sectional study to survey US clinicians about their knowledge, perceptions, and attitudes regarding awareness, testing and treatment of Lp(a). We utilized an experienced medical survey company (MedSurvey) to survey clinicians utilizing methodology that has been previously published [15,16]. MedSurvey utilizes the Centers for Medicare Services (CMS) to build their respondent panel. Participating clinicians must have an NPI number and are invited using several methodologies (phone/mail/email). Once invited and responding with their interest, they went through a thorough verification process to verify their identity before they could participate in surveys. A small stipend was provided to each clinician completing the survey. Our survey study qualified as non-human subjects research and was exempt from institution review board review but utilized a study information sheet requiring participants to verify their consent to participate. Our analysis utilized only de-identified data.

We initially targeted surveying 2000 clinicians (including 1000 cardiologists and 1000 primary care clinicians and specialists, including neurologists and endocrinologists and up to 120 nurse practitioners and physician assistants) representing all major geographic regions of the United States, rural and urban, and among private, academic, and government practices. Clinicians enrolled were required to have experience treating patients with elevated LDL-C, have been in practice for at least 5 years and seeing at least 25 patients per week, with no more than 5% classifying themselves as a lipid specialist.

### 1.2 Variables

Our survey collected information on the following: clinician characteristics, including sex, and race/ethnicity, primary medical subspecialty: cardiology, primary care (e.g., family medicine, internal medicine, OB/GYN), endocrinology, or neurology, and medical discipline: physician, nurse practitioner, and physician associate/assistant, practice location (rural, urban, suburban), and access to a lipid specialist (Y/N).

Awareness of Lp(a) as a significant risk driver for ASCVD was described in questions assessing whether the clinician agreed or not to the following statements: 1) Lp(a) is a significant risk driver for ASCVD, 2) elevated Lp(a) is the most prevalent genetic cause of ASCVD, 3) knowledge of Lp(a) levels helps me stratify risk of my patients for initiation or intensification of preventive therapies, and 4) knowledge of Lp(a) can help increase patient engagement in helping me better explain to my patients their ASCVD risk.

Current perception regarding Lp(a) testing was described in questions asking whether clinicians agreed or not about the following statements including whether Lp(a): 1) should be measured in all persons at least once (universal screening), 2) should be measured in persons who have a history of premature ASCVD, 3) should be measured in persons who have a family history of premature ASCVD, 4) should be measured in persons who have a history of any recurrent ASCVD event, 5) should be measured in persons with a recent (in the past 12 months) ASCVD event, 6) should be measured in persons with a family history of elevated Lp(a), 7) should be measured in persons with diabetes (without ASCVD), 8) should be measured in persons without ASCVD but with multiple risk factors (other than diabetes), 9) should be measured in persons with suspected or known aortic stenosis, and 10) whether they would recommend testing for all first-degree relatives if Lp(a) is elevated in a patient.

Barriers to Lp(a) testing were described by questions asking clinicians to what extent they perceived the following as barriers as to why lipoprotein(a) is infrequently measured: 1) lack of awareness about its importance as a risk driver for ASCVD, 2) lack of currently approved therapies for lowering lipoprotein(a), 3) lack of harmonized guidelines on who should be tested for lipoprotein(a), 4) lack of specific guidelines on how to treat elevated lipoprotein(a) currently, 5) lack of uniformity in measurement methods (nmol/L vs. mg/dL), and 6) difficulty to get the Lp(a) test reimbursed.

Current management approaches for ASCVD patients with elevated Lp(a) were assessed by 2 steps: step 1, a scenario was given for a patient with a prior MI and normal LDL-C and normal Lp(a) to solicit the recommendations for initiate treatment; step 2, recommendations for further treatments beyond high intensity or maximally tolerated statin if the patient above additionally had Lp(a) levels of 50 mg/dL (or 125 nmol/L), 70 mg/dL (or 175 nmol/L), 90 mg/dL (or 225 nmol/L), 120 mg/dL (or 300 nmol/L)].

Clinicians’ expectations for future agents for reducing Lp(a) were assessed by questions asking 1) what attributes they feel are the most important for a new therapy targeting lipoprotein(a), such as cardiovascular outcome data, long-term efficacy, safety, dosing frequency and mechanism of action. They were also questioned as to which patient types they agreed they would consider prescribing a lipoprotein(a)-lowering agent if shown to have cardiovascular risk reduction benefit. This included patients with 1) recurrent ASCVD event, 2) premature ASCVD event, 3) myocardial infarction, 4) ASCVD with elevated LDL-C, 5) ischemic stroke, 6) recent ASCVD event, 7) peripheral arterial disease, 8) ASCVD regardless of LDL-C, 9) without ASCVD but with other risk factors (e.g., diabetes), regardless of LDL-C, and 10) without ASCVD but with elevated LDL-C.

### 1.3 Data Analysis

Descriptive analyses were used to present survey responses overall, by clinician type and primary medical specialty. Summary statistics such as mean, standard deviation, median and interquartile range were reported for continuous variables whereas binary; categorical variables were summarized as frequencies and relative frequencies (percentages). The Chi-square test of proportions was utilized to compare proportions between two categorical variables. Multiple logistic regression was used to explore demographic and other clinician characteristics that relate to testing for lipoprotein(a) and clinicians’ attitudes regarding the significance of Lp(a) as a risk factor, and other outcomes of interest.

## 2 Results

Overall, completed surveys were received from 2002 clinician respondents between May and July 2024, of which 28.1 % were female, 58.2 % of White, 24.1 % Asian, and 17.6 % other race or ethnicity (including Black and Hispanic) (Central Illustration). The majority of respondents (94.0 %) were physicians with the remainder nurse practitioners (3.8 %) and physician assistants (2.3 %). On average, clinicians reported a mean (standard deviation [SD]) of 19.0 (9.4) years in practice with almost one third (30.9 %) reporting in practice at least 25 years and 43.3 % seeing at least 100 patients a week. While 5.0 % noted they were a lipid specialist, 55.9 % noted they had access to a lipid specialist. More than half (51.9 %) responded that they practiced in a suburban location, 48.4 % with a private medical group, 47.2 % were primary care providers, 35.1 % cardiology and the remainder endocrinology (8.8 %) or neurology (9.0 %). Respondents noted their patient mix to be approximately 20.1 % Black, 9.0 % Asian, 17.7 % Hispanic/Latina, and 47.1 % to be White or other. Cardiology and neurology clinicians were much more likely to be male, endocrinologists were most likely to consider themselves a lipid specialist, and primary care providers were least likely to practice in an academic medical center and most likely to be in a private practice (p<0.0001 for these measures across medical specialty) (Table 1).

**Table 1:** Demographic Characteristics of Survey Respondents.

	Overall, n ( %) (n=2002)	Primary Care, n ( %) (n=944)	Cardiology, n ( %) (n=702)	Endocrinology, n ( %) (n=176)	Neurology, n ( %) (n=180)	p-value
**Sex**						<0.0001
Female	563(28.1 %)	318(33.7 %)	120(17.1 %)	85(48.3 %)	40(22.2 %)	
Male	1387(69.3 %)	610(64.6 %)	559(79.6 %)	85(48.3 %)	133(73.9 %)	
Others	52(2.6 %)	16(1.69 %)	23(3.3 %)	6(3.4 %)	7(3.9 %)	
**Race/Ethnicity**						0.171
White or Caucasian	1166(58.2 %)	574(60.8 %)	404(57.6 %)	94(53.4 %)	94(52.2 %)	
Asian or Asian American	483(24.1 %)	215(22.8 %)	177(25.2 %)	42(23.9 %)	49(27.2 %)	
Others	353(17.6 %)	155(16.4 %)	121((17.2 %)	40(22.7 %)	37(20.6 %)	
**Medical discipline**						<0.0001
Physician	1881(94.0 %)	887(94.0 %)	641(91.3 %)	173(98.3 %)	180(100 %)	
Nurse Practitioner	75(3.8 %)	32(3.4 %)	41(5.8 %)	2(1.1 %)	0(0 %)	
Physician Assistant	46(2.3 %)	25(2.7 %)	20(2.9 %)	1(0.6 %)	0(0 %)	
**Years in Practice** **(mean ± SD)**	19.0±9.4	20.3±9.2	18.0±9.6	17.7±8.5	17.3±9.4	<0.0001
**Years in Practice**						<0.0001
5-<15	732(36.6 %)	289(30.6 %)	295(42.0 %)	69(39.2 %)	79(43.9 %)	
15-<25	652(32.6 %)	322(34.1 %)	206(29.3 %)	71(40.3 %)	53(29.4 %)	
25-<55	618(30.9 %)	333(35.3 %)	201(28.6 %)	36(20.5 %)	48(26.7 %)	
**Patients Seen Per Week** **(mean ± SD)**	96.5±65.3	102.4±66.1	94.1±65.8	91.6±63.5	79.4±56.0	<0.0001
**Patients Seen Per Week**						<0.0001
25-<100	1136(56.7 %)	479(50.7 %)	404(57.6 %)	117(66.5 %)	136(75.6 %)	
≥100	866(43.3 %)	465(49.3 %)	298(42.5 %)	59(33.5 %)	44(24.4 %)	
**lipid specialist**						<0.0001
Yes	101(5.0 %)	16(1.7 %)	46(6.6 %)	26(14.8 %)	13(7.2 %)	
No	1901(95.0 %)	928(98.3 %)	656(93.5 %)	150(85.2 %)	167(92.8 %)	
**Access to lipid specialist**						0.208
Yes	1120(55.9 %)	506(53.6 %)	410(58.4 %)	104(59.1 %)	100(55.6 %)	
No	882(44.1 %)	438(46.4 %)	292(41.6 %)	72(40.9 %)	80(44.4 %)	
**Practice Location**						<0.0001
Rural	220(11.0 %)	130(13.8 %)	59(8.4 %)	10(5.7 %)	21(11.7 %)	
Suburban	1038(51.9 %)	513(54.3 %)	354(50.4 %)	87(49.4 %)	84(46.7 %)	
Urban	744(37.2 %)	301(31.9 %)	289(41.2 %)	79(44.9 %)	75(41.7 %)	
**Primary practice setting**						<0.0001
Academic medical center	408(20.4 %)	107(11.3 %)	197(28.1 %)	47(26.7 %)	57(31.7 %)	
VA/Government health system	28(1.4 %)	16(1.7 %)	9(1.3 %)	1(0.6 %)	2(1.1 %)	
Health care system	573(28.6 %)	246(16.1 %)	237(33.8 %)	43(24.4 %)	47(26.1 %)	
Private medical practice	969(48.4 %)	563(59.6 %)	253(36.0 %)	81(46.0 %)	72(40.0 %)	
Other	24(1.2 %)	12(1.3 %)	6(0.9 %)	4(2.3 %)	2(1.1 %)	
**Patient race/ethnicity proportions**						
Asian or Asian American	9.0±9.6	9.2±10.4	8.7±9.5	8.3±7.1	9.1±7.7	0.5393
Black or African American	20.1±14.5	10.3±15.3	20.2±14.2	18.9±13.4	20.3±12.7	0.7141
Hispanic or Latina	17.7±14.9	18.1±15.2	17.3±14.1	18.3±15.1	16.4±13.9	0.4278
American-Indian or Alaska Native	3.0±8.9	3.0±9.3	2.6±7.4	4.5±11.3	3.4±9.9	0.1058
Native Hawaiian or other Pacific Islander	3.1±9.5	2.9±8.8	2.9±9.4	3.4±9.6	4.5±13.0	0.1814
White / Other	47.1±26.6	46.5±26.9	48.3±26.4	46.7±26.9	46.3±25.0	0.5669

p-values indicate comparisons across clinician type

### 2.1 Lp(a) Awareness

Table 2 shows responses to questions regarding Lp(a) awareness. More than 8 in 10 respondents (81.1 %) agreed, including completely agreed, that Lp(a) is a significant risk driver for ASCVD with less than half (48.0 %) agreed Lp(a) being the most prevalent genetic cause of ASCVD. The majority (76.5 % and 75.4 %) agreed that Lp(a) helped them stratify patient risk for initiation or intensification of preventive therapies, and that knowing Lp(a) can help increase patient engagement in helping better explain to patients their risks of ASCVD, respectively (Central Illustration). There were no significant differences across provider specialty.

**Table 2:** Lp(a) Awareness.

Survey item	Overall, n ( %) (n=2002)	Primary Care, n ( %) (n=944)	Cardiology, n ( %) (n=702)	Endocrinology, n ( %) (n=176)	Neurology, n ( %) (n=180)	p-value
Agreement **(agree and completely agree)** on the following Lp(a) statements across different provider type						
Lipoprotein(a) is a significant risk driver for atherosclerotic cardiovascular disease	1624(81.1 %)	706(74.8 %)	632(90.0 %)	155(88.1 %)	131(72.8 %)	0.0719
Elevated Lp(a) is the most prevalent genetic cause of ASCVD	960(48.0 %)	455(48.2 %)	347(49.4 %)	80(45.5 %)	78(43.3 %)	0.8728
Knowledge of lipoprotein(a) levels helps me stratify risk of my patient for initiation or intensification of preventive therapies.	1532(76.5 %)	692(73.3 %)	565(80.5 %)	147(83.5 %)	128(71.1 %)	0.5222
Knowledge of lipoprotein(a) levels can help increase patient engagement in helping me better explain to my patient their risks of ASCVD	1510(75.4 %)	686(72.7 %)	559(79.6 %)	133(75.6 %)	132(73.3 %)	0.7202

p-values indicate comparisons across clinician type

### 2.2 Lp(a) Testing and Screening

While only 41.4 % of respondents agreed with universal Lp(a) screening (testing at least once), the majority agreed Lp(a) should be measured in those with family history of elevated Lp(a) (74.6 %), those who have experienced a premature event (73.7 %) or family history of premature ASCVD (71.3 %) followed by persons with any recurrent ASCVD event (67.2 %), those with severely elevated LDL-C (including familiar hypercholesterolemia) (66.2 %), persons without ASCVD event but with multiple risk factors (60.2 %) and persons with a recent ASCVD event (55.6 %). 45.1 % agreed with screening persons with diabetes and 36.4 % in those with suspected or known aortic stenosis (Table 3 and Central Illustration). In multivariable logistic regression adjusted for race/ethnicity, sex, practice location, and practice setting, compared to primary care clinicians, cardiology clinicians were 1.8-fold more likely to agree on universal screening of Lp(a) and 1.6-fold more likely to agree Lp(a) should be measured in those experiencing a premature ASCVD event or in those with a family history of a premature ASCVD event (Table 4). Only about half (50.5 %) reported they would recommend testing all first-degree relatives if Lp(a) was found to be elevated in a given patient, although such recommended cascade screening was highest in cardiology (58.6 %) and lowest in neurology (37.8 %) (Table 3).

**Table 3:** Lp(a) Testing/Screening.

Survey item	Overall, n ( %) (n=2002)	Primary Care, n ( %) (n=944)	Cardiology, n ( %) (n=702)	Endocrinology, n ( %) (n=176)	Neurology, n ( %) (n=180)	p-value
Agreement (agree and completely agree) on the following statements:						
All persons should be tested at least once (universal screening)	828(41.4 %)	342(36.2 %)	340(48.4 %)	67(28.1 %)	79(43.9 %)	0.0578
If lipoprotein(a) is elevated in my patient I will recommend testing all first- degree relatives.	1010(50.5 %)	448(47.5 %)	411(58.6 %)	83(47.2 %)	68(37.8 %)	<0.0001
Agreement **(agree and completely agree)** that Lipoprotein(a) should be measured by patient type:						
persons with a family history of elevated Lp(a).	1494(74.6 %)	665(70.4 %)	563(80.2 %)	143(81.3 %)	123(68.3 %)	0.3156
persons who have experienced a premature ASCVD event.	1476(73.7 %)	672(71.2 %)	545(77.6 %)	133(75.6 %)	126(70.0 %)	0.7190
persons with a family history of premature ASCVD.	1428(71.3 %)	642(68.0 %)	535(76.2 %)	127(72.2 %)	124(68.9 %)	0.5795
persons with any recurrent ASCVD event.	1346(67.2 %)	636(67.4 %)	461(65.7 %)	127(72.2 %)	122(67.8 %)	0.9317
persons who have severely elevated LDL-C (including familial hypercholesterolemia) (e.g., ≥190 mg/dL).	1326(66.2 %)	626(66.3 %)	449(64.0 %)	117(66.5 %)	134(74.4 %)	0.7668
persons with a recent (in the past 12 months) ASCVD event.	1112(55.5 %)	541(57.3 %)	356(50.7 %)	106(60.2 %)	109(60.6 %)	0.4706
persons with documented coronary disease (e.g, significant angiographic stenosis or percutaneous intervention) without an ASCVD event.	1103(55.1 %)	101(56.1 %)	111(63.1 %)	350(49.9 %)	541(57.3 %)	<0.0001
persons without any ASCVD event but with multiple risk factors (other than diabetes).	1006(50.3 %)	462(48.9 %)	361(51.4 %)	89(50.6 %)	94(52.2 %)	0.9546
who have at least moderately elevated LDL-C (e.g., ≥130 mg/dL).	918(45.9 %)	408(43.2 %)	339(48.3 %)	75(42.6 %)	96(53.3 %)	0.4933
persons with diabetes (without any ASCVD event).	903(45.1 %)	422(44.7 %)	309(44.0 %)	80(45.5 %)	92(51.1 %)	0.8525
persons with suspected or known aortic stenosis	729(36.4 %)	345(36.6 %)	243(34.6 %)	68(38.6 %)	73(40.6 %)	0.8401

p-values indicate comparisons across clinician type

**Table 4:** Multivariable Logistic Regression of Factors Associated with Clinician Agreement on Measuring Lipoprotein(a).

Variable	Clinician Type				p-trend
	Primary Care	Cardiology	Endocrinology	Neurology	
**Lipoprotein(a) should be measured in all persons at least once (universal screening) [Agree/Completely agree]**					
Model 1	Reference	1.61(1.34-1.92)⁎⁎	1.21(0.90-1.62)	1.55(1.16-2.07)	<0.0001
Model 2	Reference	1.80(1.49-2.17)⁎⁎	1.20(0.89-1.61)	1.67(1.24-2.24)	<0.0001
**Lipoprotein(a) should be measured in persons who have experienced a premature ASCVD event [Agree/Completely agree]**					
Model 1	Reference	1.43(1.19-1.71)*	1.29(0.96-1.74)	1.09(0.82-1.47)	0.0014
Model 2	Reference	1.56(1.29-1.89)⁎⁎	1.30(0.96-1.75)	1.19(0.88-1.61)	<0.0001
**Lipoprotein(a) should be measured in persons with a family history of premature ASCVD [Agree/Completely agree]**					
Model 1	Reference	1.48(1.24-1.78**)**⁎⁎	1.10(0.82-1.48)	0.99(0.74-1.33)	0.0001
Model 2	Reference	1.63(1.35-1.98)⁎⁎⁎	1.10(0.81-1.49)	1.06(0.78-1.42)	<0.0001
**Lipoprotein(a) should be measured in persons with a family history of elevated Lp(a) [Agree/Completely agree]**					
Model 1	Reference	1.52(1.32-1.90)⁎⁎	1.53(1.14-2.06)	0.99(0.74-1.33) *	<0.0001
Model 2	Reference	1.72(1.42-2.08)⁎⁎⁎	1.51(1.11-2.05)	1.06(0.79-1.43)	<0.0001

Model 1 is unadjusted; Model 2 is adjusted for race, gender, practice location, and practice settings.

⁎ *P* < 0.05,

⁎⁎ *P* < 0.01,

⁎⁎⁎ *P* < 0.0001

**Fig. 1:**
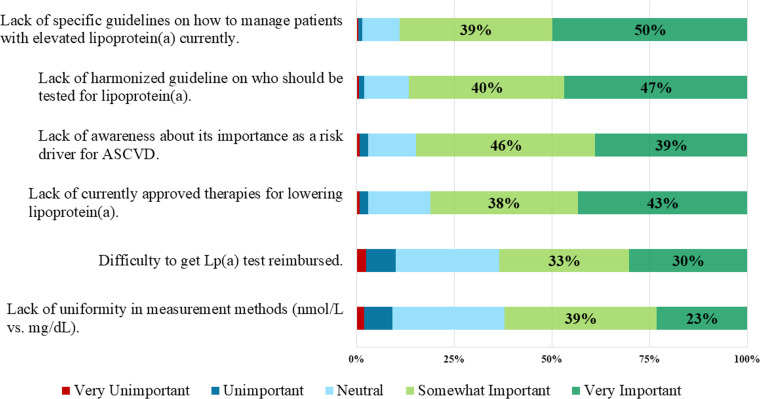
Importance of Reasons/Barriers Why Clinicians Feel Lipoprotein(a) is Infrequently Measured.

The most common reasons/barriers that clinicians deemed important (somewhat or very important) for infrequent Lp(a) testing were the lack of specific guidelines on how to manage patients with elevated Lp(a) (89.1 %), followed by lack of harmonized guidelines on who should be tested (86.6 %), lack of awareness of Lp(a) as being an important risk driver for ASCVD (84.8 %), lack of currently approved therapies for lowering Lp(a) (81.2 %), difficulty to get an Lp(a) test reimbursed (63.4 %), and lack of uniformity in measurement methods (62.1 %) (Fig. 1). Proportions responding regarding the importance of these barriers were largely similar across medical specialties, except for primary care physicians being more likely to report difficulty getting an Lp(a) test reimbursed.

### 2.3 Lp(a) treatment

When clinicians were asked about expectations in a new therapy to lower Lp(a) regarding magnitude of Lp(a) reduction expected from a new therapy, 56.1 % noted they would expect to see at least a 50 % reduction in Lp(a) levels and 13.2 % would expect at least an 80 % reduction in Lp(a) levels. In addition, 24.0 % reported they would expect to see at least a 15 % risk reduction, but 40.4 % expected to see at least a 20 % risk reduction in ASCVD events from a new therapy. There were no significant differences by clinician specialty in the proportions reporting on these attributes.

**Fig. 2:**
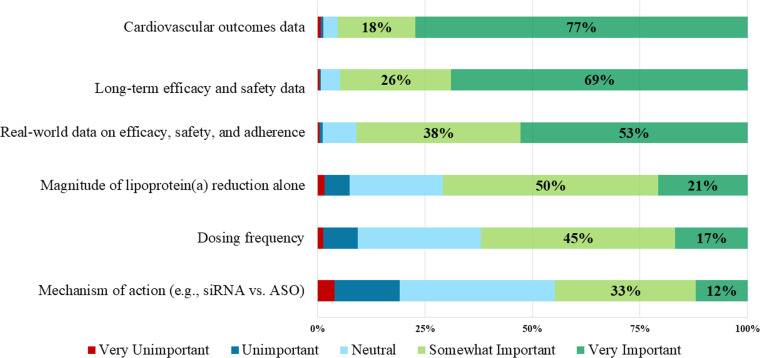
Attributes Clinicians Perceive to be Important for a New Therapy Targeting Lipoprotein(a).

As shown in Fig. 2 and the Central Illustration, the majority of the clinicians considered having cardiovascular outcomes data (77.3 %) to be very important for a new Lp(a)-targeted therapy, followed by long-term efficacy and safety data (68.9 %), real-world data on efficacy, safety, and adherence (51.9 %), magnitude of Lp(a) reduction alone (20.8 %), dosing frequency (16.9 %), and mechanism of action (e.g., SiRNA vs. ASO) (12.0 %). There were no significant differences in attributes felt to be important across provider type.

**Fig. 3:**
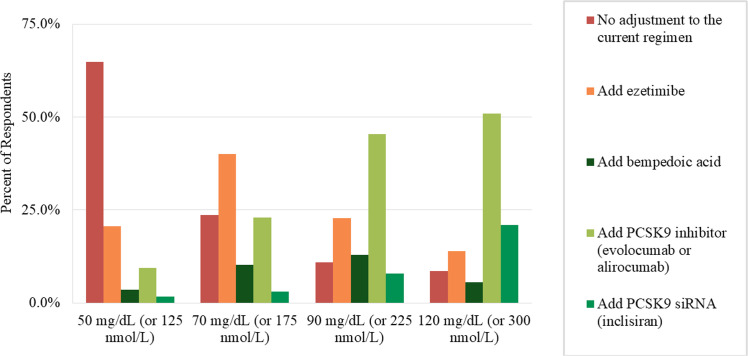
Consideration of Additional Non-Statin Therapy According to Lp(a) Thresholds.

While 25.0 % of respondents felt an Lp(a) level of 50 mg/dL (125 nmol/L) or higher would warrant more intensified treatment, proportions feeling higher thresholds were needed were 20.6 % for 70 mg/dL (175 nmol/L) or higher and 10.1 % for 90 mg/dL (225 nmol/L) or higher. In particular, 64.8 % of respondents noted they would not make changes in therapy with an Lp(a) of 50 mg/dL (or 125 nmol/L), but when this was increased to 70 mg/dL (175 nmol/L), 40.1 % indicated they would add ezetimibe. If this threshold was further increased to 90 mg/dL (225 nmol/L), 45.4 % noted they would add a proprotein convertase substilisin/kexin type 9 (PCSK9) inhibitor, and at 120 mg/dL (300 nmol/L), 50.9 % noted they would add a PCSK9 inhibitor and 21.0 % a siRNA therapy (inclisiran) (Fig. 3).

When asked of which patient types they completely agreed they would be most likely to consider prescribing Lp(a) targeted therapy if shown to have proven CVD benefit, respondents prioritized (in order of importance) patients with recurrent ASCVD events (51.0 %), premature ASCVD events (47.2 %), myocardial infarction (41.8 %), ASCVD and elevated LDL-C (38.0 %), ischemic stroke (35.6 %), recent ASCVD events (35.5 %), peripheral arterial disease (28.5 %), ASCVD regardless of LDL-C (20.7 %), without ASCVD but with other risk factors regardless of LDL-C (17.7 %), and without ASCVD but with elevated LDL-C (17.3 %). There were no significant differences across provider specialty either for most important attributes or targeted patient populations for Lp(a) therapies (Table 5).

**Table 5:** Patient Types Clinicians Agree to Consider Prescribing Lp(a) Therapy with Proven Benefit.

Survey item	Overall, n ( %) (n=2002)	Primary Care, n ( %) (n=944)	Cardiology, n ( %) (n=702)	Endocrinology, n ( %) (n=176)	Neurology, n ( %) (n=180)	p-value
Patients with recurrent ASCVD event	1020(51.0 %)	468(49.6 %)	368(52.4 %)	97(55.1 %)	87(48.3 %)	0.8647
Patients with premature ASCVD event	945(47.2 %)	420(44.5 %)	351(50.0 %)	94(53.4 %)	80(44.4 %)	0.5428
Patients with MI	836(41.8 %)	391(41.4 %)	301(42.9 %)	72(40.9 %)	72(40.0 %)	0.9775
Patients with ASCVD and elevated LDL-C	762(38.1 %)	341(36.1 %)	294(41.9 %)	71(40.3 %)	56(31.1 %)	0.3716
Patients with ischemic stroke	713(35.6 %)	329(34.9 %)	239(34.1 %)	61(34.7 %)	84(46.7 %)	0.3884
Patients with recent (e.g., past 12 months) ASCVD event	711(35.5 %)	328(34.8 %)	247(35.2 %)	69(39.2 %)	67(37.2 %)	0.9226
Patient with PAD	570(28.5 %)	256(27.1 %)	213(30.3 %)	51(29.0 %)	50(27.8 %)	0.8610
Patients with ASCVD regardless of LDL-C	414(20.7 %)	170(18.0 %)	174(24.8 %)	43(24.4 %)	27(15.0 %)	0.0888
Patients without ASCVD but with other risk factors (e.g., diabetes), regardless of LDL-C	354(17.7 %)	174(18.4 %)	116(16.5 %)	36(20.5 %)	28(15.6 %)	0.7960
Patients without ASCVD but with elevated LDL-C	347(17.3 %)	155(16.4 %)	130(18.5 %)	35(19.9 %)	27(15.0 %)	0.7723

p-values indicate comparisons across clinician type

## 3 Discussion

Our survey study of more than 2000 clinicians across the United States representing primary care, cardiology, endocrinology and neurology is the largest completed to date to examine clinician attitudes towards awareness, testing, and treatment of Lp(a). We show most clinicians agree knowing the Lp(a) level can improve risk assessment and patient engagement. Further, patients with premature or recurrent CVD events are most likely to be targeted for Lp(a) testing and for prescribing a future Lp(a)-targeted therapy if shown to have CVD benefit.

We demonstrate more than 80 % of clinicians agree Lp(a) is significant risk driver for ASCVD, and that three-fourths agreed knowing the Lp(a) level would help in risk stratification and patient engagement, despite actual reported testing rates from claims data showing much lower testing rates than our data would suggest [[10], [11], [12]]. The low testing rates could potentially be attributed to lack of guidelines as to who should be tested, which was reported by our survey respondents to be the most common barrier for not testing for Lp(a), as opposed to lack of knowledge or reimbursement being among the least reported barriers to testing. While reimbursement issues were not felt to be a major barrier in our study, the lack of uniform reimbursement for testing (e.g., while many private insurances reimburse, this is not the case for Medicaid) could be a problem for disadvantaged populations, some who are among the highest risk populations for ASCVD. While recent recommendations from the National Lipid Association have called for testing at least once in all adults [9] as well as increased advocacy efforts on testing from various professional associations, about 40 % of our survey respondents agreed with universal testing of Lp(a). Since these revised recommendations were released just prior to us conducting our survey, it is unlikely sufficient time elapsed to see the effect of these recommendations on testing rates in our survey. Those with family history of elevated Lp(a) or with a premature personal history of CVD or family history of premature CVD events were the top three patient types our survey respondents felt should be prioritized for testing. While over 80 % agreed Lp(a) to be a significant risk driver for ASCVD, only about half of respondents identified it as the most common genetic cause of ASCVD or would recommend cascade screening, indicating a need to improve clinician understanding of Lp(a) heritability that could lead to greater screening of first degree relatives. Respondents felt the most important attributes for new Lp(a)-targeted therapies were having CVD outcome data, followed by long-term efficacy/safety data. They felt they would be most likely to prescribe such therapies if shown to have CVD benefits to patients with premature or recurrent CVD events, possibly because of their perception that such patients are likely at higher risk for future events and/or elevated Lp(a) being more common in such patients. There is a need for more data on Lp(a) distribution and relation to future ASCVD events in these higher risk groups. Previously published data show the risk conferred by elevated Lp(a) is further compounded among those with a family history of ASCVD [17].

Our results can be compared to other recent survey studies in smaller or more selected clinician samples. Among 126 survey respondents to a brief electronic survey distributed select groups of University of Pennsylvania Health System (UPHS) providers, only 31 % answered that they test for Lp(a) regularly in their practice with the presence of ASCVD and a family history of ASCVD being the most common reasons for testing, consistent with our findings. More than two-thirds (69 %) noted they do not currently measure Lp(a) levels in their patients, noting lack of knowledge with Lp(a), insurance/ billing concerns, and lack of clinical trial outcomes data and pharmaceutical interventions as key barriers [18]. Moreover, among 53 lipid clinics surveyed in the United Kingdom, 81 % had access to lipoprotein(a) measurement with 60 % noting they measured Lp(a) at least once in their patients with the leading indications being a personal or family history of premature history of cardiovascular disease in those <60 years old, which also compares to our findings. 60 % also noted they performed family cascade testing when Lp(a) was found to be ≥200 nmol/L in a given patient [19]. Also, among 151 clinics that are part of the European Atherosclerosis Society (EAS) lipid clinic network 76 % of clinicians noted they routinely measured Lp(a) with the most common reasons for not testing being the lack of reimbursement, treatment options, non-availability of Lp(a) testing, and the cost of performing the test, also common issues seen in other countries that may limit testing. Among those who noted they routinely measure Lp(a), they mostly used it to further cardiovascular risk assessment, with about half recognizing 50 mg/dL (approx. 110 nmol/L) as the threshold for increased cardiovascular risk [20].

Our findings of higher thresholds of Lp(a) prompting clinicians to suggest use of non-statin therapies with ezetimibe and PCSK9 inhibitors recommended at higher levels support the findings of Shah and colleagues [21] studying electronic health records (HER) from 5 health systems where they noted those with elevated Lp(a) levels were more frequently initiated on ezetimibe or PCSK9 inhibitors although regardless of Lp(a) levels there was greater uptake of statin, ezetimibe, and PCSK9 inhibitors compared with those without a test, showing that both an Lp(a) test alone as well as higher levels of Lp(a) are actionable for initiating non-statin therapies. However, delaying consideration of non-statin therapies until Lp(a) is elevated represents gap in care if these are not being used when LDL-C alone remains suboptimal on statin therapy alone. Of interest, our results suggest higher testing rates for Lp(a) than has been reported previously from studies of health claims data where <1 % of all patients and <5 % of those with ASCVD are noted to be screened [10,11]. The inclusion criteria requiring clinicians who treat LDL-C as well as potential biases resulting from those most interested in completing a survey related to lipid management could explain these differences.

Our study has certain strengths and limitations. The large sample size and systematic screening methods utilized by a reputable medical survey company is an important strength. However, as is the case of most medical survey studies, response rates are quite limited, which may bias the sample towards those most interested and engaged in the topic of the survey (in this case, dyslipidemia). In addition, as our survey was limited to cardiology, neurology, endocrinology and primary care clinicians, we did not include all provider types where Lp(a) may be important in the disease process, such as vascular surgery/medicine. Moreover, even though the stipend paid to complete the survey was quite modest, this could have impacted and biased participation as well. In addition, survey studies such as ours are unable to validate what is actual practice (e.g., despite 41 % agreeing with universal screening, the real-world data suggest the testing rate in ASCVD patients is below 5 %). Finally, certain questions that would have provided more insight such as on whether respondents have had requests for reimbursement denied and how often, as well as how often their patients found to have elevated Lp(a) suffer emotional stress due to a lack of available therapies.

In conclusion, our US nationwide survey shows that the majority of clinicians agree Lp(a) is a significant risk driver for ASCVD and knowing the Lp(a) level can improve risk assessment and patient engagement. Further, patients with premature or recurrent CVD events are most likely to be targeted for Lp(a) testing and for prescribing a future Lp(a)-targeted therapy if shown to have CVD benefit. Our survey of Lp(a) awareness, testing, management and future therapeutics represents the most recent and comprehensive survey on this topic to date. Our results are hopefully useful to guide the prioritization of efforts to increase awareness, patient populations to be tested, as well as priorities to focus on in the development and use of newer therapeutic strategies.

## Funding

This study was supported by a contract from Novartis Pharmaceuticals to the University of California, Irvine.

## Disclosures

Dr. Wong notes research support from Amgen, Novartis, Ionis, and Regeneron, and is a consultant for Novartis and Ionis, advisory board member for Amgen, and speaker for Novartis. Drs. Ward, Schludi, and Hu are employees of Novartis.

## CRediT authorship contribution statement

**Nathan D. Wong:** Writing – review & editing, Writing – original draft, Supervision, Project administration, Conceptualization. **Yihang Fan:** Writing – original draft, Methodology, Formal analysis. **Wenjun Fan:** Writing – review & editing, Formal analysis. **Jonathan H Ward:** Writing – review & editing. **Belinda Schludi:** Writing – review & editing. **Xingdi Hu:** Writing – review & editing, Methodology, Funding acquisition.

## Declaration of competing interest

Dr. Wong notes research support from Amgen, Novartis, Ionis, and Regeneron, and is a consultant for Novartis and Ionis, advisory board member for Amgen, and speaker for Novartis. Drs. Ward, Schludi, and Hu are employees of Novartis.

## References

[1] Tsimikas S. (2017). A test in context: lipoprotein(a). J Am Coll Cardio.

[2] (2009). Emerging Risk Factors Collaboration. Lipoprotein(a) concentration and the risk of coronary heart disease, stroke, and nonvascular mortality. JAMA.

[3] Willeit P., Ridker P.M., Nestel P.J., Simes J., Tonkin A.M., Pedersen T.R., Schwartz G.G., Olsson A.G., Colhoun H.M., Kronenberg F., Drechsler C., Wanner C., Mora S., Lesogor A., Tsimikas S. (2018). Baseline and on-statin treatment lipoprotein(a) levels for prediction of cardiovascular events: individual patient-data meta-analysis of statin outcome trials. Lancet.

[4] Wong N.D., Zhao Y., Sung J., Browne A. (2021). Relation of first and total recurrent atherosclerotic cardiovascular disease events to increased lipoprotein(a) levels among statin treated adults with cardiovascular disease. Am J Cardiol.

[5] Wong N.D., Zhao Y., Xiang P., Coll B., López JAG. (2020). Five-year residual atherosclerotic cardiovascular disease risk prediction model for statin treated patients with known cardiovascular disease. Am J Cardiol.

[6] Wong N.D., Fan W., Hu X., Ballantyne C., Hoodgeveen R.C., Tsai M.Y., Browne A., Budoff M.J. (2024). Lipoprotein(a) and long-term cardiovascular risk in a multi-ethnic pooled prospective cohort. J Am Coll Cardiol.

[7] Reyes-Soffer G., Ginsberg H.N., Berglund L., Duell P.B., Heffron S.P., Kamstrup P.R., Lloyd-Jones D.M., Marcovina S.M., Yeang C., Koschinsky M.L., American Heart Association Council on Arteriosclerosis, Thrombosis and Vascular Biology; Council on Cardiovascular Radiology and Intervention; and Council on Peripheral Vascular Disease. Lipoprotein(a) (2022). A genetically determined, causal, and prevalent risk factor for atherosclerotic cardiovascular disease: A scientific statement from the American Heart Association. Arterioscler Thromb Vasc Biol.

[8] Grundy S.M., Stone N.J., Bailey A.L., Beam C., Birtcher K.K., Blumenthal R.S., Braun L.T., de Ferranti S., Faiella-Tommasino J., Forman D.E., Goldberg R., Heidenreich P.A., Hlatky M.A., Jones D.W., Lloyd-Jones D., Lopez-Pajares N., Ndumele C.E., Orringer C.E., Peralta C.A., Saseen J.J., Smith S.C., Sperling L., Virani S.S., Yeboah J. (2019). 2018 AHA/ACC/AACVPR/AAPA/ABC/ACPM/ADA/AGS/APhA/ASPC/NLA/PCNA Guideline on the Management of blood Cholesterol: A report of the American College of Cardiology/American Heart Association Task Force on Clinical Practice Guidelines. Circulation.

[9] Koschinsky M.L., Bajaj A., Boffa M.B., Dixon D.L., Ferdinand K.C., Gidding S.S., Gill E.A., Jacobson T.A., Michos E.D., Safarova M.S., Soffer D.E., Taub P.R., Wilkinson M.J., Wilson D.P., Ballantyne CM. (2024). A focused update to the 2019 NLA scientific statement on use of lipoprotein(a) in clinical practice. J Clin Lipidol.

[10] Hu X., Cristino J., Gautam R., Mehta R., Amari D., Heo J.H., Wang S., Wong ND. (2023). Characteristics and lipid lowering treatment patterns in patients tested for lipoprotein(a): A real-world US study. Am J Prev Cardiol.

[11] Bhatia H.S., Hurst S., Desai P., Zhu W., Yeang C. (2023). Lipoprotein(a) testing trends in a large academic health system in the United States. J Am Heart Assoc.

[12] Kelsey M.D., Mulder H., Chiswell K., Lampron Z.M., Nilles E., Kulinski J.P., Joshi P.H., Jones W.S., Chamberlain A.M., Leucker T.M., Hwang W., Milks M.W., Paranjape A., Obeid J.S., Linton M.F., Kent S.T., Peterson E.D., O'Brien E.C., Pagidipati N.J. (2023). Contemporary patterns of lipoprotein(a) testing and associated clinical care and outcomes. Am J Prev Cardiol.

[13] Bhatia H.S., Ma G.S., Taleb A., Wilkinson M., Kahn A.M., Cotter B., Yeang C., DeMaria A.N., Patel M.P., Mahmud E., Reeves R.R., Tsimikas S. (2022). Trends in testing and prevalence of elevated lp(a) among patients with aortic valve stenosis. Atherosclerosis.

[14] Eid W.E., Sapp E.H., Conroy C., Bessinger C., Moody C.L., Yadav R., Tolliver R., Nolan J., Francis SM. (2024). Increasing provider awareness of lp(a) testing for patients at risk for cardiovascular disease: A comparative study. Am J Prev Cardiol.

[15] Wong N.D., Bang M., Block R.C., Peterson A.L.H., Karalis DG. (2021). Perceptions and barriers on the use of Proprotein Subtilisin/Kexin type 9 inhibitors in heterozygous familial hypercholesterolemia (From a Survey of Primary Care Physicians and Cardiologists). Am J Cardiol.

[16] Block R.C., Bang M., Peterson A., Wong N.D., Karalis DG. (2021). Awareness, diagnosis and treatment of heterozygous familial hypercholesterolemia (HeFH) - results of a US national survey. J Clin Lipidol.

[17] Hedegaard B.S., Bork C.S., Kaltoft M., Klausen I.C., Schmidt E.B., Kamstrup P.R., Langsted A., Nordestgaard BG. (2022). Equivalent impact of elevated lipoprotein(a) and familial hypercholesterolemia in patients with atherosclerotic cardiovascular disease. J Am Coll Cardiol.

[18] D'Souza J., Soffer D.E., Bajaj A. (2024). Attitudes and barriers to lipoprotein(a) testing: A survey of providers at the University of Pennsylvania Health System. J Clin Lipidol.

[19] Ansari S., Neely R.D.G., Payne J., Cegla J. (2024). Lipoprotein(a) testing in lipid clinics across the UK: results of a national survey. J Clin Lipidol.

[20] Catapano A.L., Tokgözoğlu L., Banach M., Gazzotti M., Olmastroni E., Casula M., Ray K.K., Lipid Clinics Network Group (2023). Evaluation of lipoprotein(a) in the prevention and management of atherosclerotic cardiovascular disease: A survey among the Lipid Clinics Network. Atherosclerosis.

[21] Shah N.P., Mulder H., Lydon E., Chiswell K., Hu X., Lampron Z., Cohen L., Patel M.R., Taubes S., Song W., Mulukutla S.R., Saeed A., Morin D.P., Bradley S.M., Hernandez A.F., Pagidipati N.J. (2024). Lipoprotein(a) testing in patients with atherosclerotic cardiovascular disease in 5 large US health systems. J Am Heart Assoc.

